# Epidemiology and clinical presentation of community-acquired *Staphylococcus aureus* bacteraemia in children under 5 years of age admitted to the Manhiça District Hospital, Mozambique, 2001–2019

**DOI:** 10.1007/s10096-023-04580-2

**Published:** 2023-03-18

**Authors:** Marcelino Garrine, Llorenç Quintó, Sofia Santos Costa, Augusto Messa, Arsénia J. Massinga, Delfino Vubil, Tacilta Nhampossa, Sérgio Massora, Sozinho Ácacio, Anélsio Cossa, Betuel Sigaúque, Quique Bassat, Isabel Couto, Inácio Mandomando

**Affiliations:** 1 Centro de Investigação em Saúde de Manhiça (CISM), Maputo, Mozambique; 2 Global Health and Tropical Medicine, GHTM, Instituto de Higiene e Medicina Tropical, IHMT, Universidade Nova de Lisboa, UNL, Lisbon, Portugal; 3 ISGlobal, Hospital Clínic-Universitat de Barcelona, Barcelona, Spain; 4 Instituto Nacional de Saúde (INS), Ministério da Saúde, Maputo, Mozambique; 5 ICREA, Pg. Lluís Companys 23, 08010 Barcelona, Spain; 6 Pediatrics Department, Hospital Sant Joan de Déu, (University of Barcelona), 2, 08950, Barcelona, Spain; 7 Consorcio de Investigación Biomédica en Red de Epidemiología Y Salud Pública (CIBERESP), Madrid, Spain

**Keywords:** *Staphylococcus aureus*, Bacteraemia, Paediatric, Incidence, Epidemiology

## Abstract

*Staphylococcus aureus* bacteraemia (SAB) is one of the most common bloodstream infections globally. Data on the burden and epidemiology of community-acquired SAB in low-income countries are scarce but needed to define preventive and management strategies. Blood samples were collected from children < 5 years of age with fever or severe disease admitted to the Manhiça District Hospital for bacterial isolation, including *S. aureus*. Between 2001 and 2019, 7.6% (3,197/41,891) of children had bacteraemia, of which 12.3% corresponded to SAB. The overall incidence of SAB was 56.1 episodes/100,000 children-years at risk (CYAR), being highest among neonates (589.8 episodes/100,000 CYAR). SAB declined significantly between 2001 and 2019 (322.1 to 12.5 episodes/100,000 CYAR). In-hospital mortality by SAB was 9.3% (31/332), and significantly associated with infections by multidrug-resistant (MDR) strains (14.7%, 11/75 *vs.* 6.9%, 14/204 among non-MDR, *p* = 0.043) and methicillin-resistant *S. aureus* (33.3%, 5/15 *vs.* 7.6%, 20/264 among methicillin-susceptible *S. aureus*, *p* = 0.006). Despite the declining rates of SAB, this disease remains an important cause of death among children admitted to MDH, possibly in relation to the resistance to the first line of empirical treatment in use in our setting, suggesting an urgent need to review current policy recommendations.

## Background

*Staphylococcus aureus* bacteraemia (SAB) is one of the most common bloodstream infections worldwide [1, 2]. In 2019, *S. aureus* was amongst the top three pathogens responsible for global deaths associated with antimicrobial resistance (AMR), with methicillin-resistant *S. aureus* (MRSA) causing more than 100,000 annual deaths [3]. While SAB epidemiology is well described in high-income countries [2, 4], data from low-income countries, particularly in sub-Saharan Africa, remain scarce [5, 6]. This study aims to describe the burden, epidemiological trends and clinical presentation of community-acquired SAB among children aged < 5 years in southern Mozambique, between 2001 and 2019.

## Material and methods

The study was conducted at the Manhiça District Hospital (MDH) by the *Centro de Investigação em Saúde de Manhiça* (CISM). The Manhiça district, a rural area in southern Mozambique, has a subtropical climate, with a warm and rainy season (November to April) followed by a cool and drier season [7]. The geographical and socio-demographic characteristics of the study community are described elsewhere [7, 8]. CISM’s health and demographic surveillance system (HDSS) documents vital events and migrations in the Manhiça district (2,380 km^2^) with an estimated population of 201,845 inhabitants (27,560 children aged < 5 years) living in 46,441 households [8]. Since 1997, the CISM and MDH operate a 24h morbidity surveillance, which includes the collection of clinical data of all paediatric patients and a systematic collection of a single venous blood sample for bacterial isolation upon hospital admission for all children aged < 2 years, and for children aged 2 and < 15 years with axillary temperature ≥ 39 ºC or with signs of severe illness [9]. All the data analysed were obtained from the microbiological and morbidity surveillance databases of the study area [7–9]. Minimum community-based incidence rates of SAB and 95% CIs were calculated considering individual time at risk for children residing in the CISM study area excluding periods of migration. In calculating person-time, individuals were excluded during a lag period of 15 days after each episode of community-acquired bacteraemia. Negative binomial regression models were estimated to compare incidence rates. Score test for trend of rates with calendar year was assessed by Mantel–Haenszel type method. Logistic regression models were used to compare the prevalence of SAB among all admitted children. Wilcoxon rank sum tests were used for nonparametric comparisons. Nutritional status was assessed using weight-for-age z scores, calculated using the LMS method and the 2000 CDC growth reference charts [10]. In-hospital case fatality rate (CFR) due to SAB was calculated for children with known outcome, excluding patients that left the hospital without medical permission (absconders) or those transferred to Maputo Central Hospital (MCH). Data on antibiotic resistance—MRSA and multidrug-resistant (MDR) phenotypes of this *S. aureus* collection [11] were compared with clinical and epidemiological features using χ^2^ or Fisher’s exact test, considering a significance level of 5% (STATA version 17, StataCorp LP, Texas, USA).

## Results

### Study population and positivity of SAB

From January 1, 2001, to December 31, 2019; 50,293 children aged < 5 years were admitted to MDH, and blood cultures collected on admission for 83.3% (41,891) of these patients. Bacteraemia was diagnosed in 7.6% of cases (3,197/41,891) with *S. aureus* isolated in 0.9% (394/41,891) of the blood cultures, corresponding to 12.3% (394/3,197) of bacteraemic patients. The proportion of SAB was highest among children aged ≤ 28 days (31.4%, 120/382), followed by 24–59 months (10.3%, 64/620) and 29 days-11 months (10.2%, 113/1,112), and 12–23 months (8.9%, 97/1,083) (*p* < 0.0001). The rate of *S. aureus* isolation was similar among males and females (53.3%, 210/394 *vs.* 46.7%, 184/394, respectively).

### Minimum community-based incidence rates

**Fig. 1:**
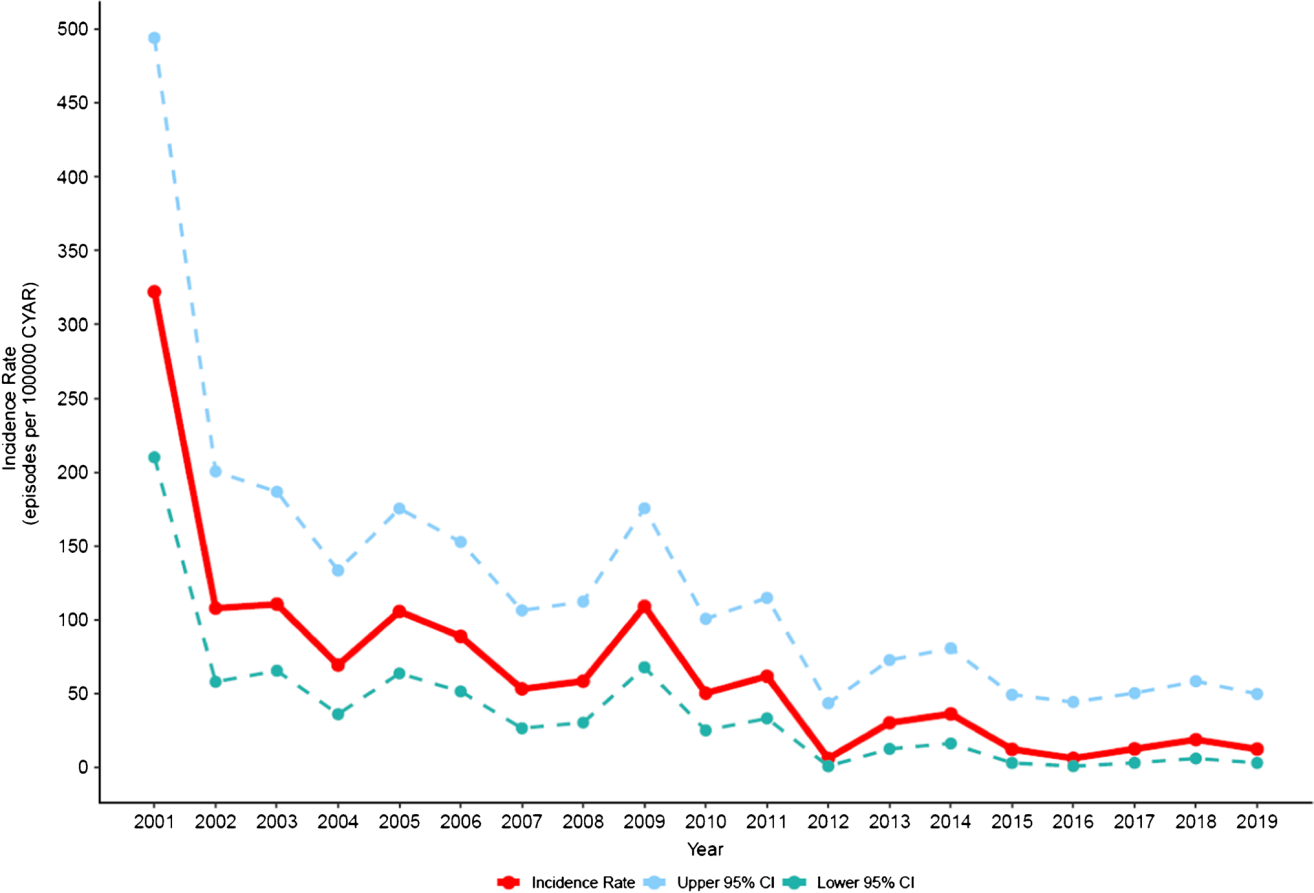
Trends of minimum estimates of community incidence rates of bacteraemia by *S. aureus* between 2001 and 2019. CYAR—Children-years at risk

Of the 394 SAB episodes, only 39.6% occurred in children living within the HDSS area, yielding an overall minimum community-based incidence of 56.1 episodes (95% CI, 47.9–65.6) per 100,000 children-years at risk (CYAR). The highest incidence was observed among children aged ≤ 28 days with 589.8 episodes (95% CI, 404.5–860.1) per 100,000 CYAR) and decreased with age (*p* < 0.0001): 92.5 episodes (95% CI, 68.8—124.3) for children aged 29 days—11 months, 89.3 (95% CI, 68.4—116.6) for 12–23 months, and 18.7 episodes (95% CI, 13.2—26.6) per 100,000 CYAR aged 24–59 months. The over-time incidence trend analysis showed a decline throughout the years, from 322.1 (95% CI, 210.0—494.1) to 12.5 (95% CI, 3.1—49.8) episodes per 100,000 CYAR between 2001 and 2019 (*p* < 0.0001), Fig. 1. The incidence rate was twice as high in the rainy season compared to dry season (77.3 [95% CI, 63.9—93.5] *vs.* 35.7 [95% CI, 27.1—47.2] episodes/100,000 CYAR, respectively;* p* < 0.0001).

### Clinical data

Complete clinical records were only available for 66.7% (263/394) of SAB patients and 72.9% (2,044/2,803) of patients with other bacteraemia (by other bacterial pathogens). Patients with SAB were significantly more likely to have clinical malaria and less likely to have non-severe and severe anaemia, fever, clinical pneumonia, and dehydration, than patients with other bacteraemia (Table 1). The measure of Lambaréné Organ Dysfunction Score (LODS) for illness severity was similar among patients with SAB and with other bacteraemia (Table 1), while the median length of stay (LOS) was higher in the second group (4 days [IQR: 3–7] *vs.* 5 days [IQR: 3–9], *p* < 0.0001; respectively).

**Table 1:** Clinical data displayed by children with *S. aureus* bacteraemia and with other bacteraemia, Manhiça District, Mozambique, 2001 – 2019

Clinical data		Other bacteraemia	*S. aureus* bacteraemia	Multivariate analysis	*p*-value^c^
		N = 2,044 (%)	N = 263 (%)	OR^a^ (95% CI^b^)	
Age (months)^d^		14.9 (12.7)	11.0 (12.4)	0.9 (0.9—0.9)^e^	< 0.0001
Clinical Malaria		686 (34)	96 (37)	1.5 (1.1—2.1)	0.0123
Severe Malnutrition (WAZ < -3)^f^		491 (24)	40 (15)	0.8 (0.5—1.1)	0.1554
Anaemia	No-Anaemia	499 (24)	102 (39)	Reference	0.0008
	Non-Severe Anaemia	1283 (63)	136 (52)	0.6 (0.4—0.8)	
	Severe Anaemia	262 (13)	25 (10)	0.5 (0.3—0.8)	
Fever		1998 (98)	248 (94)	0.4 (0.2—0.9)	0.0269
Diarrhoea		548 (27)	60 (23)	1.1 (0.8—1.6)	0.6085
Vomiting		535 (26)	52 (20)	0.8 (0.6—1.2)	0.4508
Clinical Pneumonia		1180 (58)	93 (35)	0.6 (0.4—0.8)	0.0068
Splenomegaly		547 (27)	53 (20)	0.8 (0.6—1.1)	0.1446
Dehydration		437 (21)	30 (11)	0.4 (0.3—0.7)	0.0003
LODS^g^	0	1416 (69)	206 (78)	Reference	0.1275
	1	517 (25)	50 (19)	0.7 (0.5—1.0)	
	2	99 (5)	6 (2)	0.4 (0.2—1.1)	
	3	12 (1)	1 (0)	0.8 (0.1—6.8)	

^a^OR, odds ratio; ^b^CI, confidence interval; ^c^*p*-*value* determined by Logistic regression; ^d^Arithmetic Mean (SD); ^e^determined per month increase; ^f^WAZ, weight-for-age z score, ^g^Lambaréné Organ Dysfunction Score. Non-severe anaemia is a packed cell volume (PCV) in the range ≥ 15%—< 33%, and severe anaemia as a PCV < 15%. Acute clinical malaria was defined as a child admitted with a clinical diagnosis of malaria with confirmed *P. falciparum* asexual parasitaemia > 0 parasites/μL

### In-hospital case fatality rates

Of the 394 SAB patients, 32 were transferred to MCH, 29 left MDH without medical permission, and one had no medical record. The overall CFR for *S. aureus* was 9.3% (31/332), with the highest CFR in children aged 24–59 months (20.8%, 10/48), followed by 12–24 months (9.6%, 8/83), 29 days-11 months (9.3%, 9/97), and ≤ 28 days (3.8%, 4/104) (*p* = 0.021). We did not observe significant differences by calendar year (*p* = 0.957; data not shown); or between rainy and dry season (9.0%, 21/232 *vs.* 10.0%, 10/100, respectively; *p* = 0.785). Most deaths occurred early on admission, being documented within the first (14/31, 45.2%), second (4/31, 12.9%), third or beyond day of admission (13/31, 41.9%). Three patients died on the 15 days follow up: upon being discharged (*n* = 1), transferred (*n* = 1) or absconded from the hospital (*n* = 1).

### Co-infections among patients with SAB

One hundred and six (26.9%, 106/394) SAB patients had malaria, while 3.5% (14/394) presented co-infections with other bacteria, including streptococci and Gram-negative pathogens, and three were simultaneously infected by two *S. aureus* with distinct antibiotic resistance profiles. Data of 332 children with complete medical record were analysed to compare mortality of patients with SAB + malaria to those with SAB only (9.7%, 9/93 *vs.* 9.2%, 22/239, respectively; *p* = 1.000), and among patients with SAB co-infected by other bacterial pathogens to those with SAB only (33.3%, 4/12 *vs.* 8.4%, 27/320, respectively; *p* = 0.018). The median LOS was significantly extended among patients with SAB only compared to those with SAB + malaria (5 days [IQR: 3–8] *vs.* 3 days [IQR: 2–5], respectively; *p* < 0.001), and similar between patients with SAB only and those with SAB co-infected by other bacteria (4 days [IQR: 3–7] *vs.* 4 days [IQR: 2–11], respectively; *p* = 0.926).

### Antibiotic therapy prescription

**Fig. 2:**
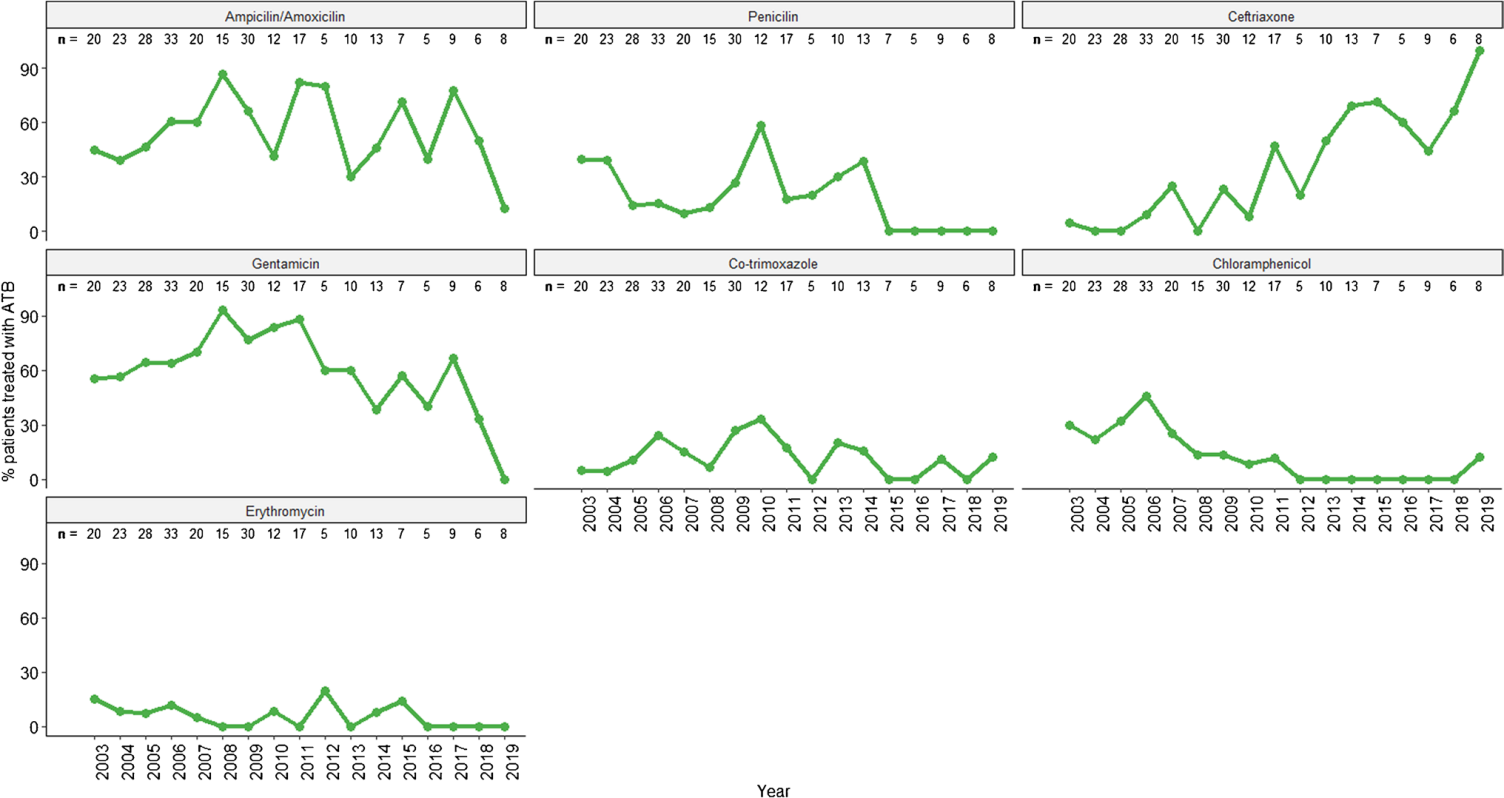
Trend of antibiotics prescribed among patients with bacteraemia by *S. aureus* admitted at the Manhiça District Hospital between 2003 and 2019. The number at the top (n) corresponds to the total of patients with bacteraemia by *S. aureus* admitted in that year

A complete record of antibiotic prescription was available for 287 (287/394, 72.8%) SAB patients, 91.6% (263⁄287) of which received antibiotics upon admission. Children treated with antibiotics were likely to present extended LOS than those not treated (5 days [IQR: 3–7] *vs.* 3 days [IQR: 2–4], respectively), although this difference was not significant (*p* = 0.163); while the mortality rate was similar (11.5%, 25/217 *vs.* 9.1%, 2/22, respectively; *p* = 1.000). Data on antibiotic prescribed were available since 2003 for 261 patients. The most administered antibiotics were gentamicin (63.9%, 167/261), ampicillin/amoxicillin (55.9%), ceftriaxone (24.5%), penicillin (21.8%), chloramphenicol (19.2%), co-trimoxazole (14.6%), and erythromycin (6.1%). The antibiotic prescription trend varied over the two decades (Fig. 2). Ceftriaxone prescription increased throughout the years, while erythromycin and chloramphenicol prescription decreased. Use of ampicillin/amoxicillin and gentamicin remained similar throughout the surveillance period, while penicillin was frequently prescribed until 2014, and prescription of co-trimoxazole oscillated throughout the study period.

### Comparison between AMR and outcome

Infections by MDR strains were likely to be associated with mortality compared to non-MDR strains (14.7%, 11/75 *vs.* 6.9%, 14/204, *p* = 0.043, respectively). Similarly, infections by MRSA were associated with mortality compared to methicillin-susceptible *S. aureus* (MSSA) (33.3%, 5/15 *vs.* 7.6%, 20/264, respectively; *p* = 0.006). The median LOS was similar between patients infected by MDR and non-MDR strains (5 days, IQR: 3–8 *vs.* 4 days, IQR: 2–7, respectively; *p* = 0.1629), but extended among patients infected by MRSA strains compared to those infected by MSSA (7 days; IQR: 3–12.5 *vs.* 4 days, IQR: 3–7), respectively), although this difference was not significant (*p* = 0.1345).

## Discussion

We described the epidemiological and clinical data of two decades of surveillance of community-acquired SAB among children admitted at MDH, showing a decline on SAB incidence in our setting. However, this incidence could be underestimated considering that only 39.6% of the cases were from the study area and identified through passive detection (severe cases for which health care was sought). This declining trend is similar to those observed for other invasive bacterial pathogens and clinical malaria previously reported in our setting [12–15]. These downward trends probably reflect the improved socioeconomic and nutritional status, improved water supply, sanitary and overall hygiene conditions, more effective prevention of mother to child HIV transmission, and better access to antiretroviral treatment by patients infected by the HIV. The highest incidence of SAB detected among neonates was previously reported in our setting [9] and recently in Gambia [16], probably reflecting the relatively immature immune responses [17].

The SAB rate observed in our study (12.3%) was similar to the one previously reported in the same setting between 2001 and 2006 [9]. However, this previous study did not evaluate the clinical characteristics and trend of bacteraemia by pathogen nor evaluated its association with malaria. In our study, SAB patients with co-infections by other bacteria were more likely to present a poor outcome, calling for an early and accurate diagnosis for a proper inpatient clinical management. The rate of SAB plus co-infections with other bacteria (3.5%) and the CFR (7.8%) observed were similar to a previous report from South Africa (7% and 8.8%, respectively), a neighbouring country of Mozambique [18]. However, the CFR reported here might be underestimated considering that more than 50% of paediatric deaths in the study area occur at home, with no previous hospital attendance [19]. The high proportion of deaths reported early on admission suggests that the children sought hospital care in critical conditions, or that the infection was caused by isolates resistant to the initial empirical antibiotic therapy. We observed an association between mortality and infection by MRSA and MDR, in addition to the extended LOS among MRSA-infected patients. These findings corroborate with previous reports showing persistent infection and high mortality among patients infected by MRSA compared to MSSA [20, 21], likely due to inappropriate empirical antibiotic therapy. Thus, identifying children with risk factors for MDR and MRSA infection will allow better guidance for empiric antibiotic therapy and a likely improved survival. In our study only a single blood culture was performed per patient, hindering the evaluation of infection persistence, although patients’ clinical evolution was reassessed every day.

The Mozambican national guidelines recommend parenteral combination of penicillin plus gentamicin or chloramphenicol (less prescribed due to its toxicity, despite occasional use in case of antibiotic stock out) for children > 2 years or ampicillin plus gentamicin as empirical antibiotic therapy in younger children or those cases of severe malnutrition. Treatment is reassessed based on clinical evolution and blood culture results using ceftriaxone for infections by MDR strains or severe disease. Our data demonstrated that these antibiotics were the most used in our setting, with ceftriaxone increasingly prescribed throughout the years. We recently reported low rates of SAB caused by MRSA or gentamicin-resistant strains (< 6%), but high for penicillin-resistant strains (90%) in our setting [11]. This resistance may challenge the empirical therapy based on combination of ampicillin/penicillin plus gentamicin, while ceftriaxone may continue as a valuable alternative. Due to the lack of complete information on treatment duration, number of doses of antibiotic given and prescription alterations, we were unable to assess the quality and appropriateness of antimicrobial therapy and relate it to patient’s outcome.

## Conclusion

In spite of the general declining rates of SAB in our population, this disease remains an important cause of death among children admitted to MDH, possibly in relation to the resistance to the first line of empirical treatment in use in our setting, suggesting an urgent need to review current policy recommendations.

## Data Availability

The datasets generated during and/or analysed during the current study are not publicly available to protect our participants’ sensitive data but are available from the corresponding author on reasonable request.
